# Can Telemedicine mitigate the High Loss-to-Follow-Up rate among Neuro-Oncology patients in Developing Countries?

**DOI:** 10.12669/pjms.41.13(PINS-NNOS).13506

**Published:** 2025-12

**Authors:** Komal Naeem, Ceemal Fareed Khan, Syed Ather Enam

**Affiliations:** 1 Komal Naeem, MD. Centre of Oncological Research in Surgery (COORS), Aga Khan University Hospital, Karachi, Pakistan; 2 Ceemal Fareed Khan, BS. Department of Surgery, Aga Khan University Hospital, Karachi, Pakistan; 3 Syed Ather Enam, MD, PhD. Centre of Oncological Research in Surgery (COORS), Aga Khan University Hospital, Karachi, Pakistan

**Keywords:** Telemedicine, Lost to Follow-Up, Neurosurgery

To the editor,

A paradigm shift in all aspects of life was witnessed during the COVID-19 pandemic. The use of technology rose exponentially in the healthcare industry during the pandemic. Telemedicine or teleconsultation, once an infrequent occurrence, became the only solution to the continuum of care in a relatively safer environment. That is when the physician community appreciated the utility of telemedicine not only in emergency but also in routine care provision.^1^

Pakistan Brain Tumor Epidemiology study has reported a staggering proportion, as high as 41.4% for loss-to-follow-up (LTFU) among neuro-oncology patients in Pakistan.^2^ On average, the patients traveled 104 km (inter-quantile range [IQR]: 9.07 - 304) to seek neurosurgical care, where almost one in every six patients (15.7%) travelled >500 km.^3^ The high LTFU rate hinders the provision of optimal health care to neuro-oncology patients which in turn negatively affects the treatment outcomes. Neuro-oncology management is multi-disciplinary and complex, entailing multiple follow-up visits to different sub-specialties and long-term surveillance.

A recent systematic review highlighted the role of telerehabilitation (from initial visits to post-operative follow-ups) in the setting of global neurosurgery. This review analyzed 40,537 patients, with a dominant representation from India. It was the only low-middle-income country included in the review. Telemedicine was identified as an efficient and cost-effective interventionas the patients reported to have good outcomes and improved health-care access. India’s cost analysis showed a saving of 61.8 USD (~17,299 PKR) per patient.^1^

Kumar et al. surveyed 250 neurosurgical patients who used audio and video-based consultation and assessed their satisfaction. Around two-thirds (208) of the patients had neuro-oncological diagnosis. They found previous exposure to WhatsApp as an important determinant of the patients’ experience. As familiarity with the application ensures the ease of use. Video consultation was recognized to have easier access, be more time-saving, and higher overall satisfaction when compared to audio teleconsultation, p<0.05. Patients perceived their concerns were better addressed in the video consultation. Nonetheless, patients highly recommended both audio and video neurosurgical consultations.^4^

Neupane et al. reported a dire incident of an infant’s demise in rural Nepal, where most of the neurosurgical centers are concentrated in urban areas. The 12-month-old had surgery done for congenital hydrocephalus. The patient suffered from a seizure upon return to the home. The child expired en route to the tertiary care center and the author argued that the provision of standardized care via teleconsultation in situations like these may improve outcomes.^5^

LTFU is a multi-factorial problem and will require multi-level mitigation strategy. Implementation of telemedicine can serve as an efficient and cost-effective solution and can potentially decrease the high LTFU rate. The lessons learned during the COVID-19 pandemic can be used while building an infrastructure for teleconsultations. Owing to the similar patient demographics the studies from India are relevant to our scenario, however, in India, internet services are available at subsidized rates which increases its penetration in remote areas. A lot of patients there have access to high-speed internet, an important requirement for tele-consultations. Laying an infrastructure will have a high upfront cost. In developing countries, like Pakistan, it is critical that the healthcare sector work with the telecommunication industry to devise telemedicine application/platform that is beneficial for the patient as well as profitable for the companies to ensure its sustenance. Simultaneously, efforts should be made to improve populations’ health and digital literacy.

In conclusion, it is vital to continue to evolve and modify the healthcare system to make it more accessible and affordable. Adopting telemedicine for the provision of follow-up care to neuro-oncology patients may not only improve patients’ access and satisfaction but also decrease travel time and overall cost and LTFU rates. Consequently, this will improve the patient’s outcomes.

## Author`s Contribution:

**KN:** Conceived the idea, data collection, manuscript drafting.

**CK:** Literature search and data collection.

**SAE:** Conceived the idea, supervision and critical review.

All authors have read and approved the final version to be published.
